# Differences in placement of calcium phosphate-hybridized tendon grafts within the femoral bone tunnel during ACL reconstruction do not influence tendon-to-bone healing

**DOI:** 10.1186/s13018-017-0583-2

**Published:** 2017-06-02

**Authors:** Hirotaka Mutsuzaki, Hiromi Nakajima, Shunsuke Nomura, Masataka Sakane

**Affiliations:** 1 Department of Orthopaedic Surgery, Ibaraki Prefectural University of Health Sciences, 4669-2 Ami, Inashiki-gun, Ibaraki 300-0394 Japan; 2 Department of Agriculture, Ibaraki University, 3-21-1 Chuo, Ami, Ibaraki 300-0393 Japan; 3 Department of Orthopaedic Surgery, Tsukuba Gakuen Hospital, 2573-1 Kamiyokoba, Tsukuba, Ibaraki 305-0854 Japan

**Keywords:** Calcium phosphate hybridization, Tendon-to-bone healing, Tendon graft placement, Anterior cruciate ligament reconstruction, Alternate soaking process

## Abstract

**Background:**

Calcium phosphate (CaP)-hybridization of tendon grafts has been shown to improve tendon-to-bone healing. The purpose of this study was to clarify the influence of different tendon graft placement methods on tendon-to-bone healing using CaP-hybridized tendon grafts in anterior cruciate ligament (ACL) reconstructions in rabbits.

**Methods:**

We compared two methods of tendon graft placement within the femoral bone tunnel: suspension of the tendon graft within the bone tunnel (suspension group) and implantation of the tendon graft coherent with the bone socket (coherence group). CaP-hybridized tendon grafts were used in both groups. Fifty-six male Japanese white rabbits were used for this study. The results of biomechanical tests (*n* = 9) and histological analyses (*n* = 5) were evaluated at 2 and 4 weeks after surgery.

**Results:**

The ultimate failure load, stiffness, stress, soft tissue remaining in bone tunnel after biomechanical testing, and direct bonding area at tendon–bone interface did not differ significantly between the suspension and coherence groups at either 2 or 4 weeks after surgery (*p* > 0.05). In both groups, the ultimate failure load, stress, soft tissue remaining in the bone tunnel, and direct bonding area at interface at 4 weeks after surgery were significantly greater than those at 2 weeks after surgery (*p* < 0.05).

**Conclusions:**

Tendon-to-bone healing in both groups progressed until the endpoint of 4 weeks. There was no influence of the CaP-hybridized tendon graft placement method on tendon-to-bone healing at 4 weeks after ACL reconstruction in rabbits. Thus, the CaP-hybridized tendon grafts were unaffected by differences in their placement within the bone tunnel and became equally anchored to the bone tunnel during the early postoperative period. The tendon graft placement method may not influence tendon-to-bone healing in ACL reconstruction when CaP-hybridized tendon grafts are used.

## Background

We have previously developed a novel technique to improve tendon-to-bone healing that used an alternating soaking process to hybridize calcium phosphate (CaP) to tendon grafts [1]. The microstructure of the CaP-hybridized tendon grafts contained low-crystalline apatite and type I collagen, and thus resembled the microstructure of the bone [2, 3]. The use of our technique prior to tendon graft implantation stimulated osteogenesis, and areas of direct bonding were observed between CaP-hybridized tendon grafts and a newly formed bone at 2–3 weeks after implantation in a rabbit anterior cruciate ligament (ACL) reconstruction model [2, 3]. In contrast, indirect bonding with fibrous connective tissue was observed after implantation of untreated tendon grafts within bone tunnels during ACL reconstruction in rabbits [2–4]. In a goat model, ACL reconstruction with CaP-hybridized tendon grafts resulted in better anterior stability and greater in situ forces under applied anterior tibial loads using a robotic-universal force/moment sensor system at 1 year after surgery compared to those with ACL reconstruction using untreated tendon grafts [5]. Furthermore, in a recent clinical trial, use of CaP-hybridized tendon grafts improved anterior knee stability and clinical scores at 2 years after surgery and reduced the percentage of bone tunnel enlargement at 1 year after surgery, relative to the outcomes with untreated tendon grafts during ACL reconstruction [6].

There are two methods for tendon graft placement within the femoral bone tunnel during ACL reconstruction. In the first method, the implanted tendon is suspended within the femoral tunnel, and its tip does not make contact with the bone socket [7]. In the second method, the implanted tendon is coherent with the bone socket [8] and its tip makes contact with the bottom of the bone socket. It is unclear whether the differences between these two tendon graft placement methods influence tendon-to-bone healing when CaP-hybridized tendon grafts are used for ACL reconstruction. Because CaP hybridization of tendon grafts enhances new bone formation in the bone tunnel [2, 3, 5], we hypothesized that CaP-hybridized tendon grafts will be unaffected by the tendon graft placement method and will become equally anchored in the bone tunnel with both methods. The present study was performed to test the above hypothesis and clarify the influence of the tendon graft placement method on tendon-to-bone healing using CaP-hybridized tendon grafts for ACL reconstruction in rabbits.

## Methods

### Graft preparation

Fifty-six skeletally immature male Japanese white rabbits (weight range 2.5–3.0 kg; age 14 weeks) were used in this study. The flexor digitorum longus tendons were obtained from the right limb of each rabbit. A doubled 25-mm-long tendon graft with a diameter of 4.2 mm was prepared, and the tibial ends of the graft were secured to each other using a 2-0 nonabsorbable suture. At the looped femoral end of the graft, a 2-0 nonabsorbable suture was tied over a stainless-steel button and passed through the loop formed by the doubled-over tendon. The CaP hybridization method was the same as that described in our previous reports [2, 3, 5, 6]. Briefly, the grafts were soaked in 100 mL of Ca solution for 30 s, and then soaked in 100 mL of NaHPO_4_ solution for 30 s, and this soaking cycle was repeated 10 times.

### Graft placement during ACL reconstruction

**Fig. 1:**
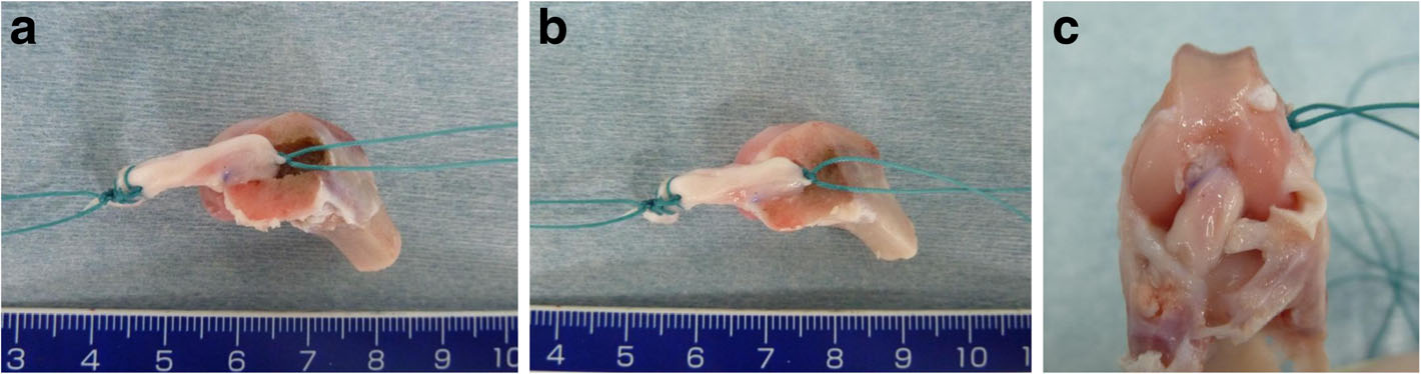
Surgical procedures shown in the *left knee* of a rabbit cadaver. **a** Suspension group: the tip of the tendon graft was placed 5.0 mm into the femoral bone tunnel. **b** Coherence group: the tendon graft was passed through the femoral bone tunnel until the tip of the tendon graft made contact with the bottom of the 5.0-mm bone socket. **c** ACL reconstruction

During surgery, the ACL was completely resected in the right knee, and a 4.2-mm-diameter femoral bone tunnel was created from the medial aspect of the lateral femoral condyle. In the suspension group, the 4.2-mm-diameter femoral bone tunnel was extended to penetrate the lateral cortex of the lateral femoral condyle. In the coherence group, the 4.2-mm-diameter femoral bone tunnel was created to a length of 5.0 mm. In the coherence group, an additional 1.2-mm-diameter bone tunnel was created from the bottom of the bone socket to the lateral cortex of the distal femur for passing of a suture during implantation of the tendon graft. Next, in both groups, a 4.2-mm-diameter tibial bone tunnel was created from the center of the tibial insertion of the ACL to the medial cortex of the proximal tibia. In the suspension group, the 5.0-mm tip of the tendon graft was passed through the femoral bone tunnel (Fig. 1a), and then secured on the femoral side with a stainless-steel button. In the coherence group, the tendon graft was passed through the femoral bone tunnel until the tip of the tendon graft made contact with the bottom of the bone socket (Fig. 1b), and then secured on the femoral side with a stainless-steel button. In both groups, the tibial end of the graft was placed in the tibial bone tunnel (Fig. 1c) and secured to the tibia with a stainless-steel button. The incision was closed with a 2-0 nonabsorbable suture. After surgery, the animals were allowed to move freely in their cages and did not receive antibiotics. At 2 and 4 weeks after surgery, nine animals in each group were euthanized by deep anesthesia for biomechanical testing, and five animals in each group were euthanized by deep anesthesia for histological analysis. These time points were selected for evaluation based on previous studies in goats and rabbits. Specifically, the biomechanical data in a CaP group and an untreated group were equal at 6 weeks after ACL reconstruction in goats [9], while tendon-to-bone healing progressed up to 4 weeks after surgery in rabbits [2, 3]. The specimens for biomechanical testing were immediately stored at −80 °C until analysis.

### Biomechanical analysis

Before biomechanical testing, each specimen was thawed for 24 h at room temperature, and the tibia was removed from the tendon graft. The cross-sectional area of the tendon graft at the joint line was measured using an apparatus specifically designed for measuring ligament cross-sectional areas (Jintai Danmenseki Sokuteiki, MEIRA Corporation, Nagoya, Japan). For biomechanical testing, each experimental femur-ACL graft complex was fixed to a custom-designed clamp that allowed tensile loading along the long axis of the graft on a single column materials-testing machine (TENSILON; STB-1225S; A&D Company Ltd., Tokyo, Japan). To ensure that we were determining the tensile properties of the graft and its interface within the tunnels, the stainless-steel button was removed. Load-to-failure testing at an elongation rate of 30 mm/min was performed, and the ultimate failure load (N) of the experimental femur-ACL graft complex was recorded. Stiffness (N/mm) was calculated from the slope of the linear region of the stress-strain curve. The ultimate failure load was divided by the cross-sectional area of the graft at the joint line to determine the stress (N/mm^2^) for each specimen.

### Histological analysis

**Fig. 2:**
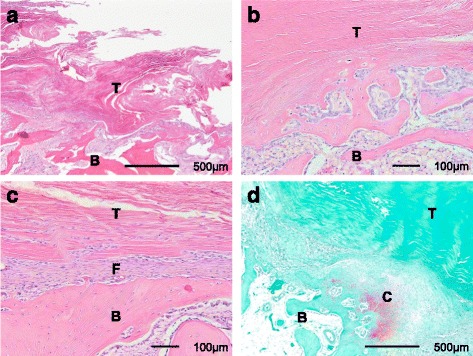
Histological sections of the tendon graft–bone interface at 4 weeks after ACL reconstruction using CaP-hybridized tendon grafts in rabbits. **a** Soft tissue remaining in the bone tunnel after biomechanical testing (H-E staining; ×40). **b** Direct bonding area at the tendon graft–bone interface (H-E staining; ×100). **c** Indirect bonding area at the tendon graft–bone interface (H-E staining; ×100). Fibrous connective tissue is visible between the tendon graft and the bone. **d** Area of *red* safranin-O-stained GAGs in the cartilage layer at the tendon graft–bone interface (×40). *T* tendon graft, *B* bone, *F* fibrous connective tissue, *C* cartilage layer

After the biomechanical testing, the specimens were fixed in 10% neutral-buffered formalin, decalcified, and embedded in paraffin. Hematoxylin and eosin (H-E) staining was performed, and the stained specimens were examined by light microscopy after staining. Using Mac Scope software (Mitani Co., Fukii, Japan), we measured the area of the soft tissue, comprising the tendon graft, and the fibrous connective tissue remaining in the bone tunnel after the biomechanical testing (Fig. 2a). The area of the soft tissue remaining in the bone tunnel was divided by the length of the bone tunnel to yield a corrected value for the remaining tendon graft. The histological failure mode for each graft (pulled out with no tendon graft remaining or midsubstance rupture with tendon graft remaining) was recorded.

The other specimens were used for histological evaluation only. After collection, the femur-ACL grafts were fixed in 10% neutral-buffered formalin, decalcified, and embedded in paraffin. H-E staining and safranin-O staining were performed prior to examination by light microscopy. The total length of direct bonding between the tendon graft and bone (the region containing cartilaginous tissue at the tendon–bone interface) was measured using Mac Scope software (Fig. 2b and c). The total length of the direct bonding interface was divided by the length of the tendon graft within the bone tunnel to determine the rate of direct bonding area at the tendon–bone interface. The area of red safranin-O-stained glycosaminoglycan (GAG) in the cartilage layer at the interface was measured using Mac Scope software (Fig. 2d). Each GAG-stained area was divided by the length of the tendon graft in the bone tunnel to give a corrected value as a width.

The ligament tissue maturation index (LTMI) described by Murray et al. [10] was used to evaluate the maturation of the tendon grafts according to the following three criteria: (1) cellular aspects including cell density, nuclear shape, and orientation; (2) extracellular matrix characteristics including crimping; and (3) vascular features including blood vessel density and maturity. The total possible score was 28 points.

### Statistical analysis

Normality of variances of the data was tested by the Shapiro–Wilk normality test. Homogeneity of variances was tested by Levene’s test when the group variances showed normality. Subsequently, group mean values were compared by Student’s *t* test when the homogeneity of variances was equal, by Welch’s *t* test when the homogeneity of variances was unequal, and by the Mann–Whitney *U* test when the group variances did not show normality. Differences were considered significant for values of *p* < 0.05. Analyses were conducted using SPSS statistical package (version 22.0; IBM Corp., Armonk, NY).

## Results

### Biomechanical testing

The results of the biomechanical testing are summarized in Table 1. There were no significant differences in the ultimate failure load between the suspension group and the coherence group at 2 weeks (*p* = 0.290) or 4 weeks (*p* = 0.566) after surgery. Within each group, the ultimate failure load at 4 weeks was significantly greater than that at 2 weeks (suspension group, *p* = 0.005; coherence group, *p* = 0.006).

**Table 1:** Biomechanical testing

	Ultimate failure load (N)	
	Suspension group (*n* = 9)	Coherence group (*n* = 9)
2 weeks	6.2 ± 3.7^+^	4.5 ± 3.0^+^
4 weeks	21.6 ± 15.1^+^	17.2 ± 10.4^+^
	Stiffness (N/mm)	
	Suspension group (*n* = 9)	Coherence group (*n* = 9)
2 weeks	10.0 ± 8.1	7.5 ± 6.6
4 weeks	15.6 ± 7.1	14.5 ± 7.0
	Stress (N/mm^2^)	
	Suspension group (*n* = 9)	Coherence group (*n* = 9)
2 weeks	0.67 ± 0.43^+^	0.48 ± 0.30^+^
4 weeks	2.35 ± 1.28^+^	1.86 ± 1.35^+^

Results are shown as mean ± SD

*:Significant difference between groups in the same week (*p* < 0.05)

+:Significant difference between weeks in the same group (*p* < 0.05)

There were no significant differences in stiffness between the suspension group and the coherence group at 2 weeks (*p* = 0.484) or 4 weeks (*p* = 0.627) after surgery. Within each group, there was no significant difference in stiffness between 2 and 4 weeks after surgery (suspension group, *p* = 0.058; coherence group, *p* = 0.052).

There were no significant differences in stress between the suspension group and the coherence group at 2 weeks (*p* = 0.310) or 4 weeks (*p* = 0.438) after surgery. Within each group, the stress at 4 weeks was significantly greater than that at 2 weeks (suspension group, *p* = 0.003; coherence group, *p* < 0.001).

### Histological analysis

The results of the histological analysis are summarized in Table 2. The corrected values for the soft tissue remaining within the bone tunnel after the biomechanical testing did not differ significantly between the suspension group and the coherence group at 2 weeks (*p* = 0.464) or 4 weeks (*p* = 0.691) after surgery. In each group, the corrected value for the soft tissue remaining within the bone tunnel at 4 weeks was significantly greater than that at 2 weeks (suspension group, *p* = 0.029; coherence group, *p* = 0.001).

**Table 2:** Histological analysis

	Failure mode (number)	
	Suspension group (*n* = 9)	Coherence group (*n* = 9)
2 weeks	PO: 2/MS: 7	PO: 2/MS: 7
4 weeks	PO: 0/MS: 9	PO: 0/MS: 9
	Soft tissue remaining within the bone tunnel after biomechanical testing (μm^2^/μm)	
	Suspension group (*n* = 9)	Coherence group (*n* = 9)
2 weeks	77.2 ± 49.3^+^	104.0 ± 95.5^+^
4 weeks	596.4 ± 586.7^+^	434.6 ± 280.5^+^
	Direct bonding area (%)	
	Suspensor group (*n* = 5)	Coherence group (*n* = 5)
2 weeks	32.1 ± 12.5^+^	30.2 ± 9.9^+^
4 weeks	51.4 ± 7.4^+^	50.9 ± 7.0^+^
	Width of GAG-stained area (μm^2^/μm)	
	Suspension group (*n* = 5)	Coherence group (*n* = 5)
2 weeks	96.1 ± 12.2	76.2 ± 50.4
4 weeks	143.1 ± 90.3	124.3 ± 33.4
	LTMI score (points)	
	Suspension group (*n* = 5)	Coherence group (*n* = 5)
2 weeks	14.4 ± 1.3	14.4 ± 0.9
4 weeks	14.8 ± 1.8	14.2 ± 0.8

Results are shown as mean ± SD

*PO* pull out, *MS* tendon midsubstance, *LTMI* ligament tissue maturation index, *GAG* glycosaminoglycan

*:Significant difference between groups in the same week (*p* < 0.05)

+:Significant difference between weeks in the same group (*p* < 0.05)

The failure mode did not differ significantly between the suspension group and the coherence group at 2 weeks (*p* = 1.000) or 4 weeks (*p* = 1.000) after surgery. At 2 weeks, seven of the nine specimens in each group failed at the tendon midsubstance, while in the remaining two specimens, the graft appeared to have pulled out of the femoral bone tunnel. At 4 weeks, all specimens in both groups failed at the tendon midsubstance.

The direct bonding area at the tendon–bone interface did not differ significantly between the suspension group and the coherence group at 2 weeks (*p* = 0.796) or 4 weeks (*p* = 0.908) after surgery. Within each group, the direct bonding area at the tendon–bone interface at 4 weeks was significantly greater than that at 2 weeks (suspension group, *p* = 0.018; coherence group, *p* = 0.005).

The width of the GAG-stained area at the tendon–bone interface did not differ significantly between the suspension group and the coherence group at 2 weeks (*p* = 0.416) or 4 weeks (*p* = 0.675) after surgery. Within each group, there was no significant difference in the width of the GAG-stained area at the tendon–bone interface between 2 and 4 weeks after surgery (suspension group, *p* = 0.311; coherence group, *p* = 0.113).

The LTMI scores did not differ significantly between the suspension group and the coherence group at 2 weeks (*p* = 0.917) or 4 weeks (*p* = 0.516) after surgery. The maturation of the tendon grafts was similar in the suspension and coherence groups at both 2 and 4 weeks after surgery. Within each group, there was no significant difference in the LTMI scores between 2 and 4 weeks after surgery (suspension group, *p* = 0.676; coherence group, *p* = 0.724).

## Discussion

In our study, the differences in placement of CaP-hybridized tendon grafts between the suspension and coherence groups did not influence the tendon-to-bone healing outcomes at 4 weeks after ACL reconstruction in rabbits. The results of the biomechanical testing and histological analysis did not differ significantly between the two groups at either 2 or 4 weeks after surgery. In rabbits, CaP-hybridized tendon grafts were shown to enhance new bone formation within the femoral bone tunnel in ACL reconstructions for up to 4 weeks after surgery [2, 3]. In the present study, CaP-hybridized tendon grafts bonded well to the femoral bone tunnel when implanted using both tendon graft placement methods examined. Yamazaki et al. [11] reported no significant effects of graft-tunnel diameter disparities of up to 2 mm on ultimate failure load at 3 or 6 weeks after ACL reconstruction in dogs. However, the conditions used in the present study were more severe than those used by Yamazaki et al. because of the shorter graft length in the bone tunnel and the shorter evaluation period.

In both groups, tendon-to-bone healing progressed until our final measurement point at 4 weeks after surgery. The ultimate failure load, stress, soft tissue remaining in the bone tunnel after biomechanical testing, and direct bonding area at the tendon–bone interface at 4 weeks after surgery were significantly greater than those at 2 weeks after surgery in both groups. The increases in the direct bonding area, ultimate failure load, and stress were likely related to the increased time after surgery. The increase in the amount of soft tissue remaining in the bone tunnel after mechanical testing can be considered to reflect the anchoring strength of the direct bond between the tendon graft and the bone, which exceeded the strength of the tendon graft. Previous studies reported the results of pull-out tests after implantation of untreated tendon grafts in the bone tunnels in rabbits and dogs [4, 12]. In these studies, the progressive increase in pull-out strength was correlated with tendon-to-bone healing [4, 12]. As the types of experimental animals and surgical methods in the previous studies differed from those in the present study, their results cannot be compared directly with our findings. However, the results of our study and the previous studies demonstrated that the tendon–bone interface progressively healed with time after graft implantation, regardless of whether untreated or CaP-hybridized tendon grafts were used.

While the width of the GAG-stained area at the tendon–bone interface and the stiffness at 4 weeks were larger than those at 2 weeks in both groups, there were no significant between-group differences in these values. The GAGs in the cartilage layers at ligamentous insertions mainly resist tensile and shear stresses [13–15]. In previous studies, CaP-hybridized tendon grafts promoted a more cartilaginous layer at the tendon–bone interface compared with the untreated group from 6 months to 2 years after ACL reconstruction in goats [5, 16–18]. A longer follow-up period is needed to evaluate the correlation between the formation of cartilaginous tissue and the stiffness at the tendon–bone interface in ACL reconstructions using CaP-hybridized tendon grafts.

The appearance of the grafted tendons was similar in the two groups. Therefore, the maturation of the CaP-hybridized tendon grafts after surgery was similar in the two groups. The tendon graft placement method also had no influence on the maturation of the CaP-hybridized tendon grafts at 4 weeks after ACL reconstruction in rabbits. The maturation of CaP-hybridized tendon grafts may continue to progress for more than 4 weeks after surgery. In addition, the grafts in two of the nine specimens in each group failed by pulling out from the femoral bone tunnel histologically at 2 weeks postoperatively. At 4 weeks after surgery, all specimens in both groups failed in the tendon midsubstance histologically. In previous reports [4, 12], all tendon grafts failed macroscopically by pulling out of the bone tunnel from 2 to 8 weeks after surgery in dogs and at 3 weeks after surgery in rabbits. As we soaked the whole tendon grafts in the CaP solutions in this study, the CaP hybridization may influence the mechanical properties of the tendon grafts.

Clinically, our results suggest that when CaP-hybridized tendon grafts are used in ACL reconstruction, it is not necessary to change the postoperative rehabilitation protocols during the early postoperative period based on the tendon graft placement method because equal tendon-to-bone healing occurs regardless of the placement method used. In addition, mismatches between the tendon graft and the bone tunnel diameter may not influence tendon-to-bone healing when CaP-hybridized tendon grafts are used.

The present study had several limitations. Bonding of the tendon–bone interface may differ between animals and humans because of between-species differences in osteogenesis. In addition, postoperative evaluations were only performed in the early postoperative period after ACL reconstruction in this study. A longer follow-up time is needed to clarify the long-term effects of the tendon graft placement methods that we tested. We also did not compare the CaP-hybridized tendon grafts with untreated tendon grafts. However, the effectiveness of CaP-hybridized tendon grafts was previously demonstrated and compared with untreated grafts for up to 4 weeks after ACL reconstruction in rabbits [2, 3].

## Conclusions

The tendon-to-bone healing in both groups progressed until the study endpoint at 4 weeks after surgery. There was no influence of the tendon graft placement method on the tendon-to-bone healing at 4 weeks after ACL reconstruction with CaP-hybridized tendon grafts in rabbits, as evaluated by biomechanical testing and histological analysis. The CaP-hybridized tendon grafts anchored equally in the bone tunnel after both placement methods, likely through enhancement of osteogenesis by the CaP hybridization. The tendon graft placement method may not influence tendon-to-bone healing in ACL reconstruction when CaP-hybridized tendon grafts are used.

## Abbreviations

ACL: Anterior cruciate ligament
CaP: Calcium phosphate
GAG: Glycosaminoglycan
